# Red Snappers

**DOI:** 10.3201/eid1510.090221

**Published:** 2009-10

**Authors:** Erin E. McConnell

**Affiliations:** Wright State University, Dayton, Ohio, USA

for a moment pretend
you are not
the infallible house staff,
but the latest admission—
hacking putrid sputum
from your soulful depths
or your festering chest,
depending on your mood.
slapped with a mask,
you are secured in secluded rooms; a paucity of guests,
but for the parade of absurd birds—
plastered in Haz-mat
lemon-yellow gowns,
and peach-colored beaks.  your meager dried-up sleep
is aborted by
bloodhungry fowl
covetous of mucus
you no longer produce.
your meals grow cold
waiting for you
in the anteroom of
your negative pressure purgatory.

